# Culture and Real-time Polymerase Chain reaction sensitivity in the diagnosis of invasive meningococcal disease: Does culture miss less severe cases?

**DOI:** 10.1371/journal.pone.0212922

**Published:** 2019-03-13

**Authors:** Sara Guiducci, Maria Moriondo, Francesco Nieddu, Silvia Ricci, Elisa De Vitis, Arianna Casini, Giovanni Maria Poggi, Giuseppe Indolfi, Massimo Resti, Chiara Azzari

**Affiliations:** 1 Department of Health Sciences, Section of Pediatrics, University of Florence, Florence, Italy; 2 Pediatric Clinic 2, Pediatric Immunology, Meyer Children’s Hospital, Florence, Italy; 3 Department of Interdisciplinary Pediatrics, Section of Medical Pediatrics, Meyer Children's Hospital, Florence, Italy; Universita degli Studi di Parma, ITALY

## Abstract

**Background:**

Invasive meningococcal disease (IMD) is a highly lethal disease. Diagnosis is commonly performed by culture or Realtime-PCR (qPCR).

**Aims:**

Our aim was to evaluate, retrospectively, whether culture positivity correlates with higher bacterial load and fatal outcome. Our secondary aim was to compare culture and qPCR sensitivity.

**Methods:**

The National Register for Molecular Surveillance was used as data source. Cycle threshold (CT), known to be inversely correlated with bacterial load, was used to compare bacterial load in different samples.

**Results:**

Three-hundred-thirteen patients were found positive for *Neisseria meningitidis* by qPCR, or culture, or both; 41 died (case fatality rate 13.1%); 128/143 (89.5%) blood samples and 138/144 (95.8%) CSF were positive by qPCR, 37/143 (25.9%) blood samples and 45/144 (31.2%) CSF were also positive in culture. qPCR was 3.5 times (blood) or 3.1 times (CSF) more sensitive than culture in achieving a laboratory diagnosis of IMD (OR 24.4; 95% CI 12.2–49.8; p < .10^−4^; Cohen’s κ 0.08 for blood and OR 49.0; 95% CI 19.1–133.4; p<10^−4^; Cohen’s κ 0.02; for CSF). Positivity of culture did not correlate with higher bacterial loads in blood (mean CT 27.7±5.71, and CT 28.1±6.03, p = 0.739 respectively in culture positive or negative samples) or in CSF (mean CT 23.1±4.9 and 24.7±5.4 respectively in positive or negative CSF samples, p = 0.11).CT values in blood from patients who died were significantly lower than in patients who survived (respectively mean 18.0, range 14–23 and mean 29.6, range 16–39; p<10^−17^). No deaths occurred in patients with CT in blood over 23. Positive blood cultures were found in 10/25 (40%) patients who died and in 32/163 (19.6%) patients who survived, p = 0.036, OR 2.73; 95% CL 1.025–7.215), however 60% of deaths would have remained undiagnosed with the use of culture only.

**Conclusions:**

In conclusion our study demonstrated that qPCR is significantly (at least 3 times) more sensitive than culture in the laboratory confirmation of IMD. The study also demonstrated that culture negativity is not associated with lower bacterial loads and with less severe cases. On the other side, in patients with sepsis, qPCR can predict fatal outcome since higher bacterial load, evaluated by qPCR, appears strictly associated with most severe cases and fatal outcome. The study also showed that molecular techniques such as qPCR can provide a valuable addition to the proportion of diagnosed and serotyped cases of IMD.

## Introduction

*Neisseria meningitidis* is a major etiologic agent of bacterial meningitis and one of the most important causes of sepsis, meningitis and other invasive bacterial diseases [1–2]. Invasive meningococcal disease (IMD) is a serious infectious disease with high morbidity and mortality rates. Diagnosis is commonly performed by culture methods or by the Real-time Polymerase Chain Reaction (qPCR).

Studies performed in different countries demonstrated that qPCR is significantly more sensitive than culture in the diagnosis of IMD [3–6]. In England 54–57% of cases were confirmed by qPCR only [3–4], in Italy that percentage is 58% [5], in Greece, in a study comparing culture and qPCR in the ability to detect pathogens causing meningitis, over 75% of cases were detected only by PCR [7]. Similar results were obtained in Delhi, India [8] where qPCR allowed serotyping of large numbers of culture negative samples so that authors concluded that molecular techniques are “of paramount importance in epidemiological surveillance especially in developing countries like India”.

However, at present it is not known whether cases lost to diagnosis in settings where only cultural tests are routinely performed are the least severe ones and/or those associated with atypical presentation.

Compared to qPCR, culture needs larger volume of sample in order to get an optimal bacterial yield and obtain bacterial growth [9] and it might be hypothesized that samples with higher bacterial load have the highest probability to be found positive in culture. Since high bacterial load is usually associated with more severe cases and poorer outcome, it could be inferred that culture can identify the most severe cases. However occasional observations in our previous studies [5–6] conflicted with that hypothesis.

Real-time PCR can give both quantitative and semi-quantitative results [10–11]. The cycle threshold (CT) value is the qPCR cycle number (of 40) at which the measured fluorescent signal exceeds a calculated background threshold, identifying amplification of the target sequence. Cycle threshold is inversely correlated with bacterial loads and it is commonly used as a proxy of bacterial load. CT can therefore be used to compare bacterial loads in different samples and as an indicator of disease severity both in IMD and in other bacterial diseases [12–16].

Therefore the first aim of the present work was to evaluate whether culture positivity correlates with higher bacterial loads measured by qPCR and/or with greater disease severity leading to a fatal outcome. Our second aim was to compare qPCR and culture sensitivity in the diagnosis of IMD in a pediatric hospital setting.

## Patients and methods

### Study design and case definition

This study evaluated retrospectively all patients with IMD caused by *Neisseria meningitidis* (sepsis or meningitis) included in the molecular surveillance register from 2007 to 2016. The register was started in 2006 in the Immunology and Infectious Disease Laboratory of the Meyer Children’s University Hospital (Florence, Italy) and was subsequently expanded with dedicated funds from the Italian Center for Disease Control (CCM) aimed to improve molecular diagnosis of invasive bacterial infections. The register was initially focused on pediatric cases: pediatric hospitals in Italy were invited to participate. Upon request by clinicians, samples obtained from adults were accepted and tested with real time PCR (qPCR) and included in the register. The number of adult patients increased over the years. Molecular surveillance was organized and is still active as a voluntary surveillance. All samples submitted to Meyer University Hospital (blood, cerebrospinal fluid, post mortem samples or other normally sterile fluids), were accompanied by clinical, demographic and outcome (death versus survival) data on the patients. The study was approved by the review board of the Department of Health Sciences. All samples included in this study had been collected as part of the routine clinical activity, and were analyzed anonymously. For that reason, a specific approval by the local ethics committee (Tuscany Regional Pediatric Ethical Committee) was not required.

The reporting of a culture-based test was welcome but not required for a case to be included in the register, so the results of culture tests were not available for all patients. Samples for molecular tests were sent to Meyer Children’s University Hospital by using a fast freepost carrier. Within 2 hours of delivery, 200 μL of sample were used for molecular diagnosis and serotyping by qPCR. Culture tests were performed by local laboratories at the original hospitals, following the hospitals’ own procedures.

Meningitis was clinically suspected in the presence of previously described signs [17]. Sepsis was clinically suspected in the presence of a compatible clinical syndrome and abnormal chemical test results [18]. A case was considered confirmed in the presence of positive culture or molecular tests [5].

### Diagnostic criteria

Laboratory confirmed diagnosis of IMD was made in the presence of qPCR positive for *ctrA* gene, or culture positivity for *Neisseria meningitidis* or both, as previously described [6]. Quantification of the *N*. *meningitidis* bacterial load was performed using the capsular transfer gene (*ctrA*), which has been shown to be specific for *N*. *meningitidis*. *CtrA* is a single-copy gene, the number of copies measured is equivalent to the bacterial load number in a range of 10^3^−10^9^ copies/mL [14]. The CT is the value at which a fluorescent signal exceeds the background indicating amplification. If there was no increase in fluorescent signal before the 40^th^ cycle, the sample was assumed to be negative.

Meningococcal serogrouping was performed by qPCR according to previously published procedures [6] and according CDC procedures (http://www.cdc.gov/meningitis/lab-manual/chpt10-pcr.html).

### Statistical analysis

Data was processed with SPSSX v.15.0: p<0.05 was considered to be statistically significant. The χ^2^ test or Fisher test was used to assess group differences in categorical variables. Odd ratios and 95%

confidence intervals (CIs) were calculated when possible. For continuous variables, Student’s test was used. Cohen’s κ and McNemar’s tests were also used for comparison of sensitivity.

The STARD 2015 (updated list of essential items for reporting diagnostic accuracy studies; http://www.equator-network.org/reporting-guidelines/stard/) has been followed.

## Results

One-hundred and eighteen hospital from 18/20 Italian Regions participated in the molecular surveillance project (S1 and S2 Figs). The two regions that did not participate in the surveillance represent, together, 0.7% of the Italian population. Patients with invasive meningococcal infections were recruited from 85 hospitals. Clinical and demographic data are shown in Table 1.

**Table 1:** Clinical and demographic data of patients included in the study.

Number of patients		313
Number of samples	Blood samples	221
	Cerebrospinal fluid samples	3
	Post-mortem specimens	2
	Synovial fluid	199
Sex	Males	161 (51.4%)
	Females	152 (48.5%)
Age range (years)		0.1–89.3
Distribution of patients in age classes	0–5 years	25 (8.0%)
	5–10 years	50 (16.0%)
	11–20 years	119 (38.0%)
	>20 years	119 (38.0%)
Distribution of patients according to clinical presentation at hospital admission	Sepsis	35 (11.2%)
	Sepsis and meningitis	183 (58.5%)
	Meningitis with no mention of sepsis	3 (1.0%)
	Arthritis	2 (0.6%)
	Pneumonia	2 (0.6%)
	Unknown	88 (28.1%)
Antibiotic treatment before microbiological tests	Yes	170 (54.3%)
	No	9 (2.9%)
	Unknown	134 (42.8%)*

*84/134 (62.7%) had received the first dose of antibiotic just before sample was taken

Since the absence of data regarding culture-based test report was not an exclusion criterion for a case to be included in the molecular surveillance register, data on culture-based tests were not available for all patients; in details: data were available for 188 of the 199 blood samples and for 146 of the 221 CSF samples.

All 313 patients were found positive for *Neisseria meningitidis* by qPCR or culture or both. In particular, all of them were found positive by qPCR (in CSF or blood or other normally sterile fluids or in more than one sample): 181/199 (91.0%) were positive in blood, 215/221 (97.3%) were positive in CSF, and 5 were positive in other materials (3/5 taken post-mortem); 42/188 (22.3%) blood cultures and 46/147 (31.3%) CSF cultures were positive.

qPCR allowed serogrouping in 307/313 patients; 6 patients were not serogrouped for sample scarcity. Serogroup distribution was the following: 189/307 serogroup B (61.6%), 95/307 serogroup C (30.9%), 12/307 serogroup W (3.9%), 11 serogroup Y (3.6%).

### Sensitivity of qPCR versus culture in blood or CSF

In order to compare sensitivity of qPCR and cultures, we included in the analysis all the samples that had been drawn at the same time to be tested for both qPCR and culture.

One hundred and forty-three blood samples were available for the study. Among them 128/143 (89.5%) were positive by qPCR and 37/143 (25.9%) were positive by culture. All the 15 cases that were negative by qPCR in blood had been admitted to hospital with the clinical suspicion of meningitis only, with no mention of sepsis, and all of them were found positive in CSF by qPCR. Details are shown in Table 2.

**Table 2:** Sensitivity of qPCR versus culture in blood or CSF.

Samples	Positive in qPCR n (%)	Positive in culture	Identified by qPCR only	Identified by culture only	
Blood n = 143	128/143 (89.5%)	37/143 (25.9%)	91/143 (63.6%)	0/143 (0.0%)	OR 24.4; 95% CI 12.2–49.8; p < .10^−4^ by χ^2^ test; Cohen’s κ 0.08; p<10^−4^ by McNemar’s test
CSF n = 144	138/144 (95.8%)	46/144 (31.9%)	92/144 (63.9%)	1/144 (0.7%)	OR 49.0; 95% CI 19.1–133.4; p<10^−4^ by χ^2^ test; Cohen’s κ 0.02; p<10^−4^ by McNemar’s test

On blood samples, qPCR was 3.5 times more sensitive than culture and qPCR was significantly more sensitive than culture in achieving laboratory diagnosis of IMD (Odds ratio 24.4; 95% CI 12.2–49.8; p < .10^−4^ by χ^2^ test; Cohen’s κ 0.08; p<10^−4^ by McNemar’s test).

As for CSF, 144 samples, taken simultaneously for both qPCR and culture, were available.

Among them 138/144 (95.8%) were positive by qPCR and 46/144 (31.9%) were positive by culture. Details are shown in Table 2. On CSF samples, qPCR was 3.1 times more sensitive than CSF culture and was significantly more sensitive than culture in achieving laboratory diagnosis of IMD (Odds ratio 49.0; 95% CI 19.1–133.4; p<10^−4^ by χ^2^ test; Cohen’s κ 0.02; p<10^−4^ by McNemar’s test).

Culture positivity rate was not different in different ages or in different Italian regions.

### Correlation between bacterial load in blood and positivity of blood cultures

In order to evaluate whether culture positivity correlates with higher bacterial loads, we included in that analysis all the samples that were positive in blood with qPCR and for which culture tests had been performed (n = 128). Blood culture was positive in 37/128 (28.9%) while blood samples were negative in culture in 90/128 (70.3%) and one sample (0.78%) was reported as contaminated by *Streptococcus viridans*.

**Fig 1:**
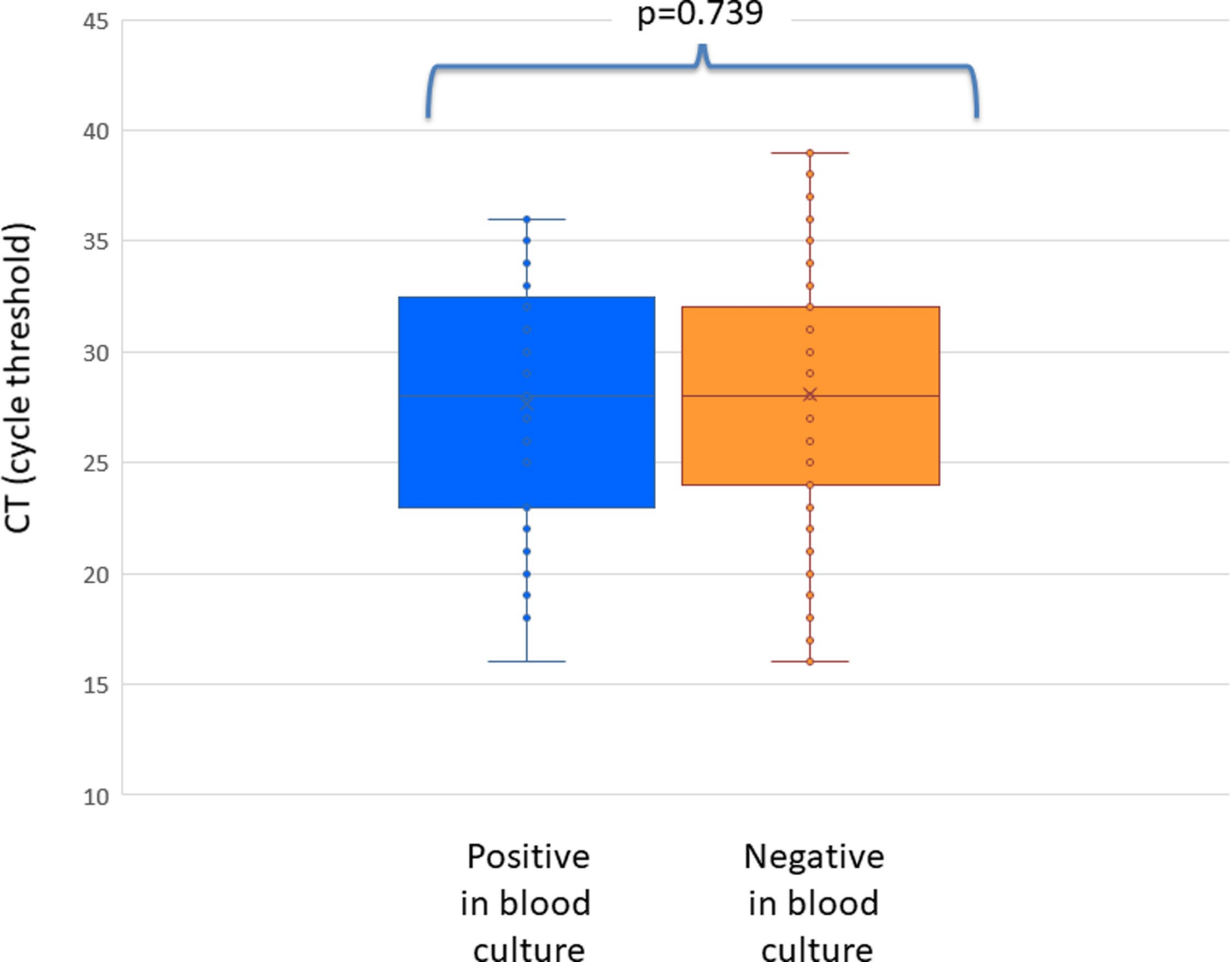
Cycle threshold in patients with invasive meningococcal disease who were found positive by qPCR in blood and who were positive (blue) or negative (orange) in blood culture. Cycle threshold is inversely proportional to bacterial load.

Positivity of blood culture did not correlate with higher bacterial loads. Actually, no difference in blood bacterial load was found between culture positive (37 samples, mean CT 27.7; SD 5.71, median 28 range 16–36) and culture negative samples (91 samples, mean CT 28.1; SD 6.03, median CT 28.0; range 16–39, p = 0.739). As shown in Fig 1, a great overlap in bacterial load values was found between culture positive and culture negative samples.

Bacterial load was not significantly different between patients who had received antibiotic treatment or not (mean CT respectively 29.3±5.7 vs 27.7±5.1; p = 0.23) and it did not differed among ages (S3A Fig).

The rate of culture positivity progressively decreased in parallel of treatment duration (S4A Fig) and among the patients who were still positive in blood after >2 days of antibiotic treatment there were patients with bacteremic meningococcal pneumonia and patients who died after complicated courses.

### Correlation between bacterial load and positivity of CSF culture

In order to evaluate correlation between culture positivity in CSF and CSF bacterial loads, we evaluated all samples that were positive by qPCR in CSF and in which culture tests had been performed (n = 138). CSF culture was positive in 45/138 (32.6%), negative in 92/138 (66.7%) and reported as contaminated in 1/138 (0.72%).

**Fig 2:**
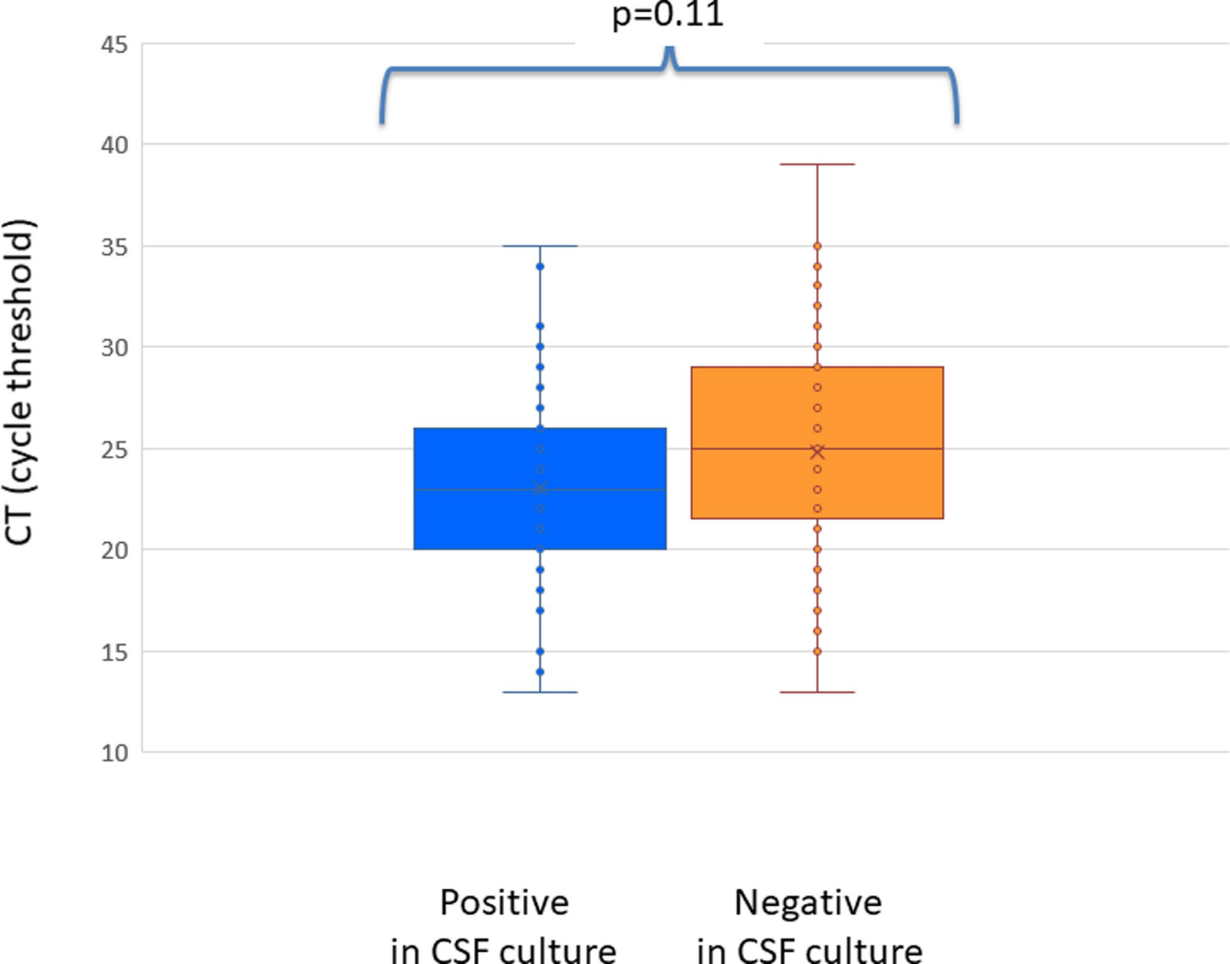
Cycle threshold in patients with invasive meningococcal disease who were found positive by qPCR in cerebrospinal fluid (CSF) and who were positive (blue) or negative (orange) in CSF culture. Cycle threshold is inversely proportional to bacterial load.

No difference was found between CSF CT in culture-positive or culture negative patients: among the 45 patients whose CSF culture was positive, CT of qPCR ranged between 13 and 35 (mean = 23.1; median = 22.5, SD 4.9); of 93 samples that tested negative for *Neisseria meningitidis* by CSF culture, CT ranged between 13 and 39 (mean = 24.7; median = 25, SD5.4); p = 0.11. (Fig 2).

Bacterial loads were not different between patients who received antibiotic treatment before hospital admission and patients who did not.

Different from what was shown in blood culture, the positivity rate of CSF cultures did not decrease in the first 2 days of antibiotic treatment (S4A Fig). However the number of patients in whom lumbar puncture was performed after 2 or more days of treatment was too low to allow stratification and statistical analysis of the results.

Differences in culture positivity among ages are shown in S3B Fig.

### Correlation between fatal outcome and bacterial load or culture positivity

#### Blood

During the study period, a total of 41 deaths occurred among the 313 patients affected by invasive meningococcal disease, resulting in a case-fatality rate of 13.1%.

In order to evaluate whether culture positivity was associated with more severe cases and fatal outcome, we evaluated all culture results from deceased patients.

The proportion of positive blood cultures in patients who died (10 positive among 25 cultures performed in patients who died; 40.0%) was higher compared to the proportion found in patients who survived (32 positive among 163 cultures performed in patients who survived 19.6%, p = 0.036, OR 2.73; 95% CL 1.025–7.215), however 60% of deaths would have remained undiagnosed with the use of culture only.

**Fig 3:**
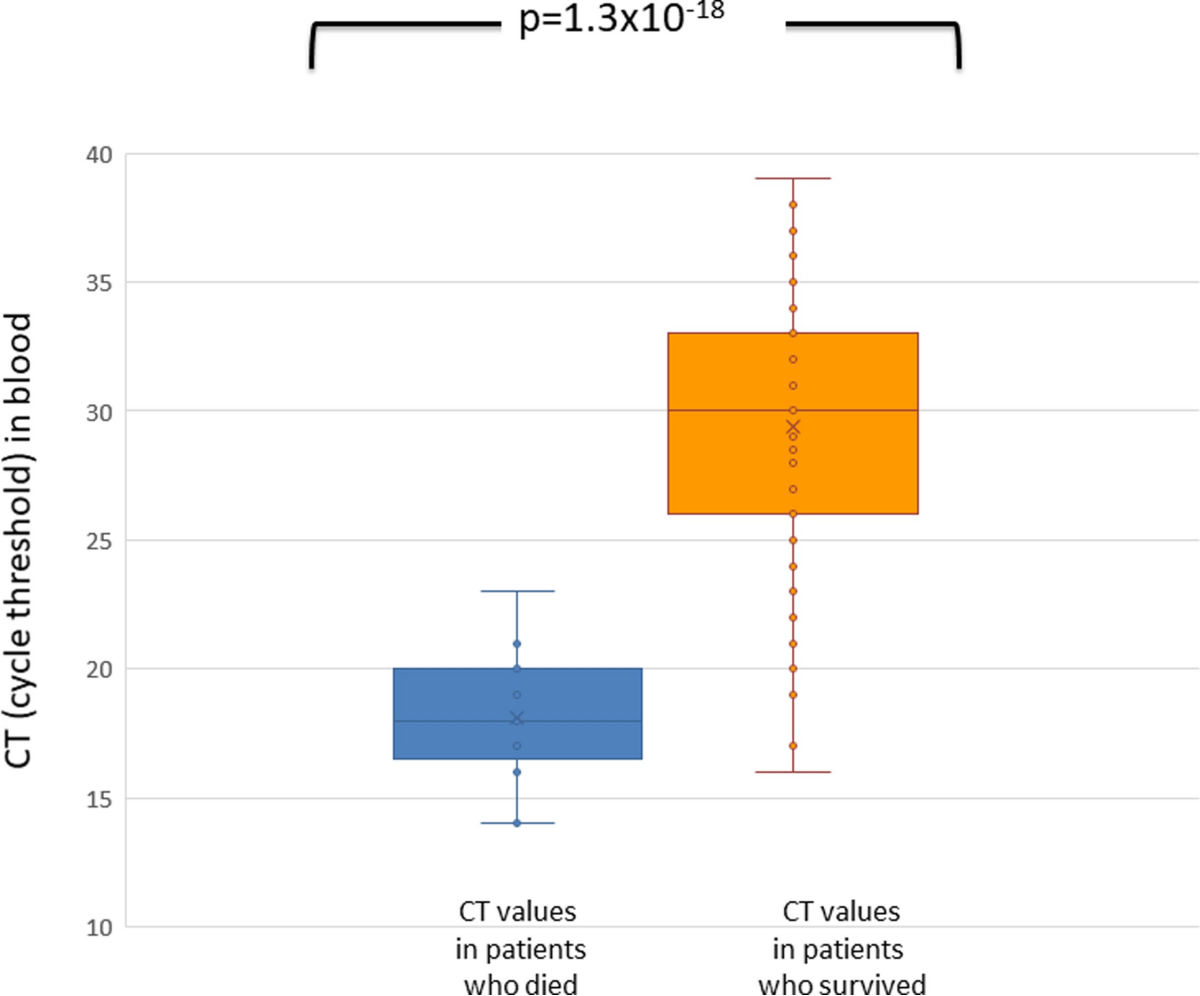
Cycle threshold in blood of patients with invasive meningococcal disease who died (blue) or survived the disease (orange). Cycle threshold is inversely proportional to bacterial load.

qPCR was able to predict fatal outcome: CT values in blood from patients who died were significantly lower, identifying higher bacterial loads, compared with CT values of patients who survived (respectively mean 18.0, median 18.0 range 14–23 versus mean 29.6, median 30 range 16–39; p<10^−17^) (Fig 3). None of the patients with CT>23 in blood had a fatal outcome. Three out of four patients who survived despite a CT < 20 had been previously vaccinated.

#### Cerebrospinal fluid

**Fig 4:**
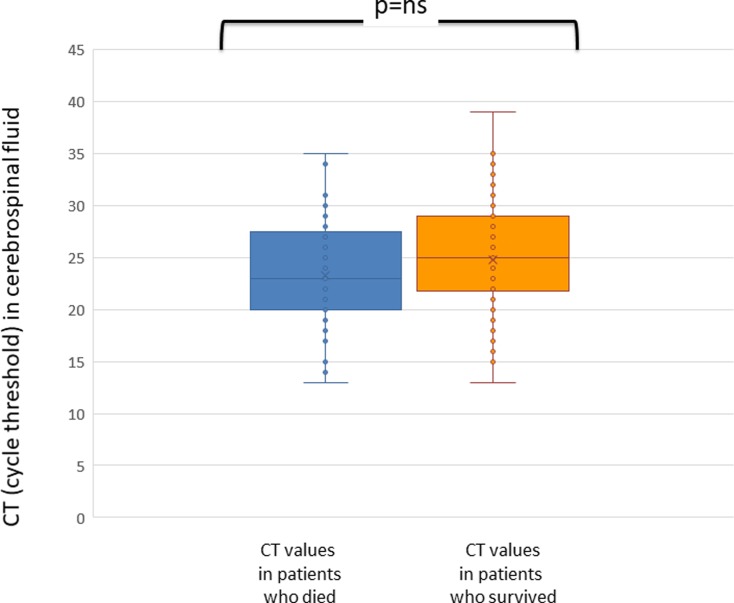
Cycle threshold in cerebrospinal fluid (CSF) of patients with invasive meningococcal disease who died (blue) or survived the disease (orange). Cycle threshold is inversely proportional to bacterial load.

Neither bacterial load nor CSF culture positivity correlated with fatal outcome. Actually, in CSF no difference was found between CT found in patients who died and CT found in patients who survived (respectively mean 24.3, range 14–35 versus mean 24.7, range 13–39, p = 0.816) (Fig 4).

As for cultures, no difference in the proportion of positive CSF cultures was found between patients who died (3/11 available cultures; 27.3%) and patients who survived (42/136 available cultures; 30.9%; p = 1.0 by Fisher test, OR 0.839, 95% CI 0.167–3.731).

Overall in 41 patients who died, blood cultures were obtained in 25 and were positive in 10/25, while CSF cultures were obtained in 11 patients and were positive in 3/11. Altogether among 41 patients who died 32 had at least one culture test performed and in 11/32 (34.4%) cultures identified an IMD; diagnoses was obtained by PCR only in 65.6% of patients who died. The proportion of patients that were identified by PCR only did not differ between patients who died (21/32, 65.6%) and patients who survived (121/184, 65.8%; p = 0.99, OR 1.006, 95% CI 0.423–2.361).

## Discussion

Several factors are known to be associated with IMD outcome: among these meningococcal serotypes and bacterial load (measured with qPCR) or host-related factors such as IL-1 and IL-1RN polymorphisms [14].

It has been previously demonstrated in different countries and setting in the world, that at least half of IMD diagnoses are missed if only culture is used for diagnosis [3,5,7,19]. However, until now, it had still been unclear whether culture might be able to identify all or at least the majority of most severe cases, whether culture positivity might be associated with higher bacterial loads and, therefore, might predict a more serious outcome of IMD.

Real-time PCR is a quantitative or semi-quantitative test and it has been previously demonstrated that bacterial load is inversely related to CT[10,14].

Our study, performed on a large group of IMD cases obtained from the national register for molecular surveillance of bacterial disease in Italy, confirmed [15] that the bacterial load was significantly higher in the blood of patients who died compared to patients who survived. Actually, low CT levels in blood strongly correlated with fatal outcome and no death was recorded in patients with CT over 23, with a positive predictive value for death of about 60% for CTs <23 and negative predictive value of 100% for CTs over that level. As for culture, our study showed that the proportion of culture-positive results is higher in patients who died compared to patients who survived IMD (40% vs 20%), but still, 60% of deaths would have remained undiagnosed with the use of culture only.

As for CSF, neither a higher bacterial load nor CSF culture positivity was significantly associated with death. That was not unexpected since it is well known that, in both IMD and pneumococcal infections, lethality is strictly associated with the presence of sepsis more than with meningitis [15, 20].

In the clinical field, qPCR is a pivotal tool not only for IMD diagnosis and serotyping but also for disease prognosis. As for diagnosis, qPCR was over 3 times more sensitive than culture in diagnosis of IMD. Furthermore, it decreases the time of identification of *N*. *meningitidis*, thus improving case ascertainment by confirmation of infections and allowing both the best therapy for the patient and prophylaxis for contacts to be initiated. Besides that, qPCR allows serogroup determination in culture negative, clinically suspected IMD and that is important for outbreak individuation, for epidemiological surveillance of IMD [21] and for vaccination planning. The thought that culture methods misses a limited number of cases and especially the less severe or atypical ones may have driven the decision not to implement molecular methods in clinical microbiology laboratories. Our study confirms that in the absence of qPCR 2/3 of etiologic diagnoses of IMD are missed and suggests, for the first time, that even among the most severe and fatal cases, 60% would remain undiagnosed. Therefore qPCR confirms to be a paramount tool in IMD surveillance. Therefore, to add qPCR to other microbiological tests as a mandatory test, would significantly improve the understanding of the disease.

Moreover a confirmed diagnosis of meningococcal infection allows antibiotic treatment spectrum and duration to be limited. In contrast, treatment to cover suspected bacterial meningitis or other invasive infections where no pathogen is identified is often much longer, with a broader range of agents used.

On the other hand, even though antibiotic resistance is not currently a problem in meningococcal infections, at present culture testing should always be performed in patients with meningitis or sepsis since it remains the most important method in acquiring information about antibiotic resistance in infections caused by to other pathogens.

It is well known [21–22] that low sensitivity of cultures is associated with antibiotic treatment started before microbiological tests; however, other factors undoubtedly interfere with the lack of sensitivity.

Actually, in the present study, most patients were admitted to the hospital without any previous antibiotic treatment and microbiological tests were performed before treatment was started. Furthermore, patients with positive or negative culture tests did not differ in the use of antibiotics or treatment (length of treatment and number of doses). Moreover, bacterial loads were not different between patients who received antibiotic treatment before hospital admission and patients who did not, as previously demonstrated in CSF [20]. Therefore, other factors, such as transport conditions or sample volume, should also be considered as important interfering factors.

An association between culture positivity and case-fatality rate has been recently found by other researchers, even though limited to pediatric cases [3]. It has been hypothesized [3] that culture positivity may be dependent on an uncontrolled bacterial replication (and consequently a higher bacterial load) or on the fact that strains found in culture may be more virulent. Our data suggest that is not the case in Italy where blood bacterial loads and severe outcome are strictly associated, while the association with blood culture positivity was poor. Since a correlation between bacterial loads and culture positivity was found in CSF and not in blood, it is difficult to imagine that strain-dependent factors are determinants of culture-positivity.

The present study has a potential bias since it was started as a pediatric study and adult patients were included in the study upon clinician request; therefore age distribution does not exactly reflect IMD age distribution in Italy, with a percentage of cases over 15 of 48% instead of an expected rate of 55%. However since no difference was found in the present study between pediatric or adult cases in term of PCR sensitivity or other study aims, it is unlikely that the lower number of adults studied may influence the study findings.

Another potential bias is that while the qPCR assays were done in a single laboratory under stringent quality control procedures, blood culture results came from the many different hospitals where the study patients were seen and no information is available about the procedure used. However no difference in culture positivity rates were found in the present study among different Italian regions so that it seems unlikely that differences among procedures may have influenced the overall results.

## Conclusions

In conclusion our study demonstrated that qPCR is significantly (at least 3 times) more sensitive than culture in the laboratory confirmation of IMD. We also demonstrated that culture negativity is not associated with lower bacterial loads and with less severe cases. Actually, even among the group of patients who died about 60% of patients would not have had laboratory confirmation without the use of qPCR. Bacterial load, evaluated by qPCR, appears strictly associated with disease outcome, since higher bacterial load were strongly associated with most severe cases and fatal outcome. Moreover our study highlighted the importance of PCR testing in confirming the diagnosis of IMD and in identifying meningococcal serotype even in culture-negative samples. Therefore, in settings with high rates of culture-negative results [5,23], molecular techniques such as qPCR can provide a valuable addition to the proportion of diagnosed cases of IMD.

## Supporting information

S1 Fig Italian regions participating in the study (in green). The only two Regions that did not recruit any patient represent, together, 0.7% of Italian population.
(TIFF)

S2 Fig Geographical distribution of hospitals participating in the study. (TIFF)

S3 Fig Meningococcal bacterial load expressed as cycle treshold (CT) in blood (panel A) or cerebrospinal fluid (panel B) in patients of different age classes. Statistical differences among ages are indicated over the bars.
(TIFF)

S4 Fig Rate of culture positivity in blood (A) or CSF (B) according to antibiotic treament duration. Number of cases are shown on the bars.
(TIFF)
