# Supplementary figures and images for: Culture and Real-time Polymerase Chain reaction sensitivity in the diagnosis of invasive meningococcal disease: Does culture miss less severe cases?
Source: PLoS One. 2019 Mar 13;14(3):e0212922. doi: 10.1371/journal.pone.0212922 (PMC6415896; doi:10.1371/journal.pone.0212922)

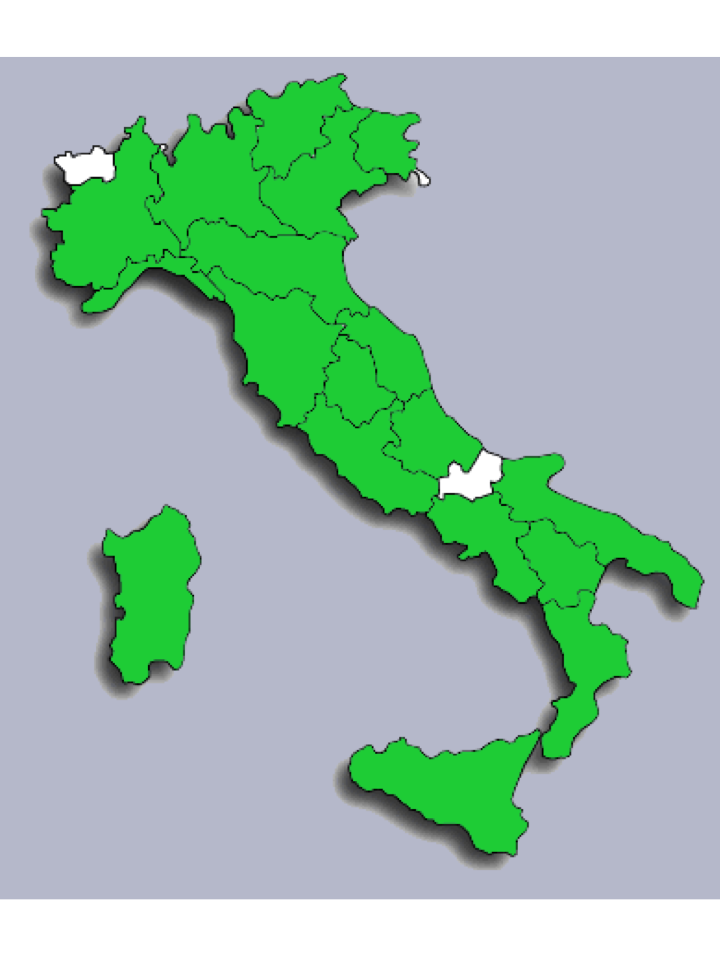

Supplement: S1 Fig — The only two Regions that did not recruit any patient represent, together, 0.7% of Italian population. (TIFF) [file pone.0212922.s001.tiff]

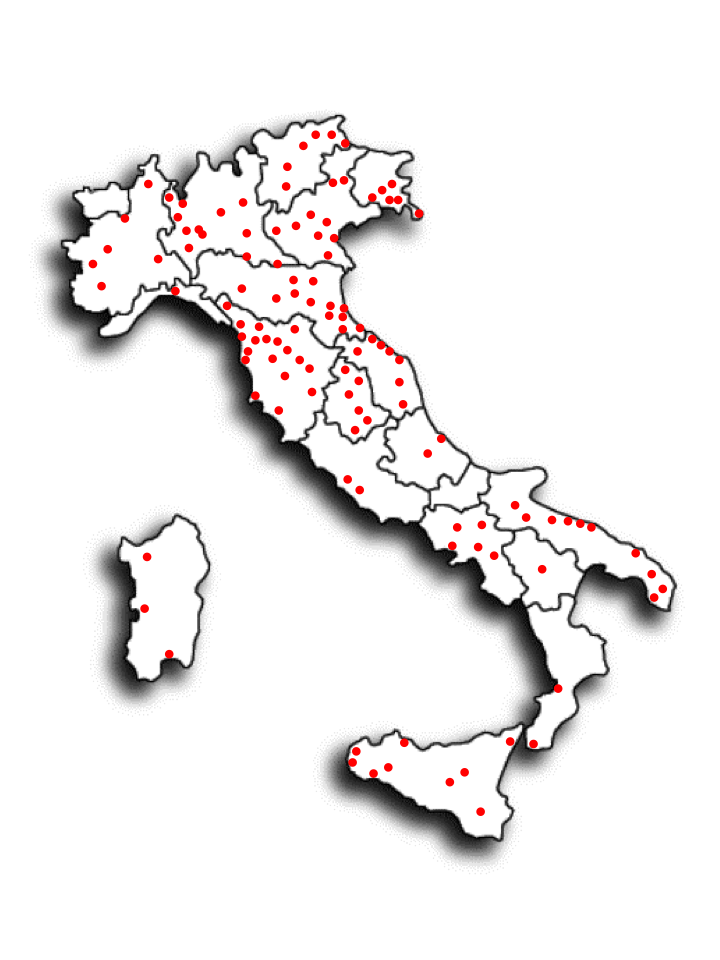

Supplement: S2 Fig — (TIFF) [file pone.0212922.s002.tiff]

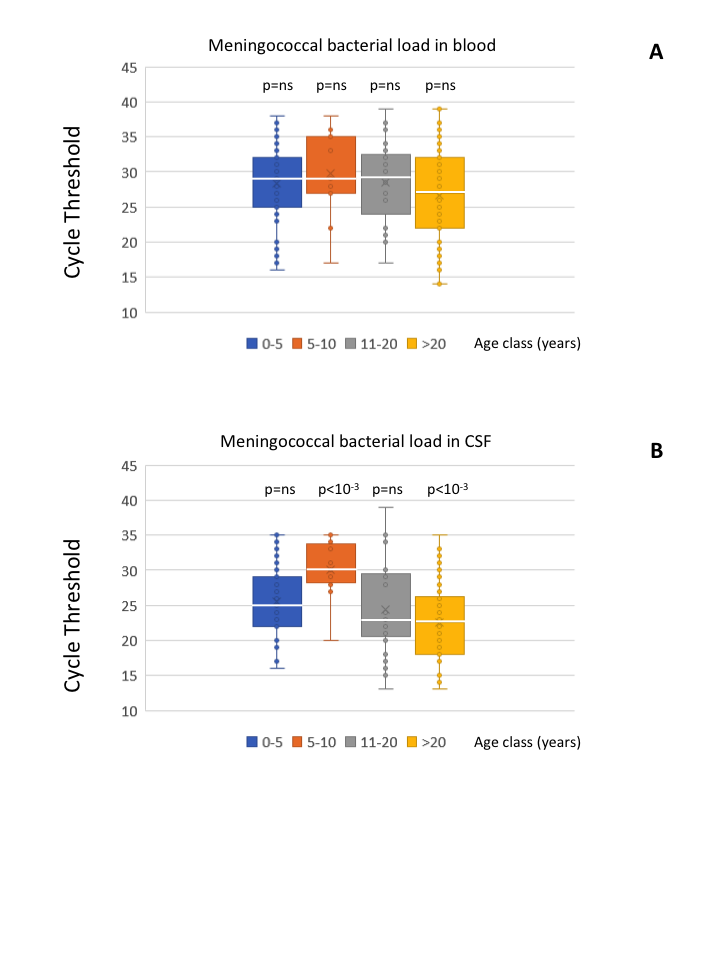

Supplement: S3 Fig — Meningococcal bacterial load expressed as cycle treshold (CT) in blood (panel A) or cerebrospinal fluid (panel B) in patients of different age classes. Statistical differences among ages are indicated over the bars. (TIFF) [file pone.0212922.s003.tiff]

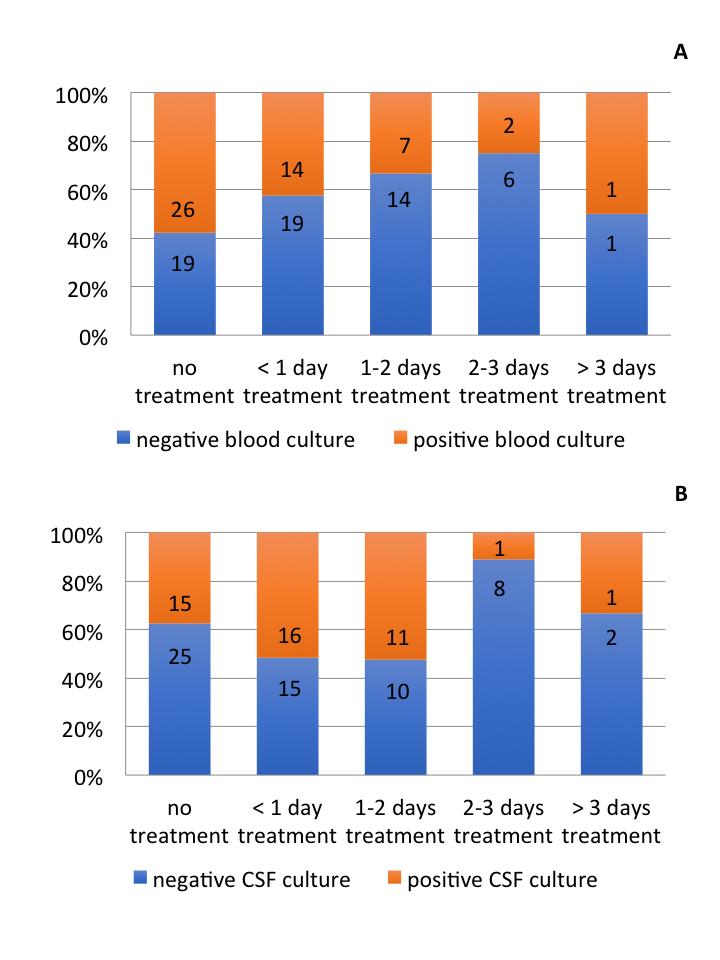

Supplement: S4 Fig — Rate of culture positivity in blood (A) or CSF (B) according to antibiotic treament duration. Number of cases are shown on the bars. (TIFF) [file pone.0212922.s004.tiff]
